# ‘From activating towards caring’: shifts in care approaches at the end of life of people with intellectual disabilities; a qualitative study of the perspectives of relatives, care-staff and physicians

**DOI:** 10.1186/s12904-015-0030-2

**Published:** 2015-07-25

**Authors:** Nienke Bekkema, Anke J. E. de Veer, Cees M. P. M. Hertogh, Anneke L. Francke

**Affiliations:** NIVEL, Netherlands Institute of Health Services Research, P.O. Box 1568, 3500 BN Utrecht, The Netherlands; Department of General Practice & Elderly Care Medicine, EMGO Institute for Health and Care Research, VU University Medical Center, P.O. Box 7057, 1007 MB Amsterdam, The Netherlands; Department of Public and Occupational Health, EMGO Institute for Health and Care Research, VU University Medical Center, P.O. Box 7057, Amsterdam, 1007 MB The Netherlands; Expertise Center for Palliative Care Amsterdam, VU University Medical Center, Amsterdam, The Netherlands

**Keywords:** End-of-life care, Intellectual disabilities, Professionals-family relationship, Values

## Abstract

**Background:**

Professionals and relatives increasingly have to deal with people with intellectual disabilities (ID) who are in need of end-of-life care. This is a specific type of care that may require a different approach to the focus on participation that currently characterizes the care for people with ID.

This paper describes the shifts in care approaches and attitudes that relatives and professionals perceive as the death of a person with ID approaches, as well as the values underlying these shifts.

**Methods:**

A qualitative design was used to reconstruct the cases of twelve recently deceased people with ID. Relatives and professionals who were closest to the person at the end of their life were interviewed. Interviews were transcribed verbatim and data were analyzed inductively, using elements of thematic analysis.

**Results:**

Five shifts were found: 1) adapting to a new strategy of comforting care, taking over tasks and symptom relief, 2) interweaving of emotional and professional involvement, 3) stronger reliance on the joint interpretation of signals expressing distress and pain, 4) magnified feeling of responsibility in medical decisions, 5) intensified caring relationship between ‘two families’: relatives and care staff.

Six relational values were behind these shifts: ‘being there’ for the person with ID, ‘being responsive’ to the person’s needs, ‘reflection’ on their own emotions and caring relationships, ‘attentiveness’ to the ID person’s wishes and expressions of distress, ‘responsibility’ for taking joint decisions in the best interests of the person, and ‘openness to cooperation and sharing’ the care with others.

**Conclusions:**

End-of-life care for people with ID involves curtailing expectations of participation and skill acquirement, and an increase in teamwork featuring intensified comforting care, symptom management and medical decision making. Three caring relationships need to be fostered: the relationship with the person with ID, relationships among professionals and the relationship between relatives and professionals. ID care services should invest particularly in the emotional support and expertise level of care staff, and in the collaboration between relatives and professionals.

## Background

In line with the ongoing process of the de-institutionalization of people with Intellectual Disabilities (ID), the focus is increasingly on the self-reliance of people with ID and their participation in society [1, 2]. Individuals with ID are characterized by significant limitations, from before the age of 18, in both intellectual functioning and adaptive behaviour [3]. Many policy and healthcare initiatives are undertaken to foster equal opportunities for people with ID, endorsed by the UN’s stated principles of equal rights for people with disabilities so that they can live, participate and be fully included in the community [1]. People with ID are encouraged to acquire new social and practical skills and to live as independently as possible [4]. Several methods are used to enhance social inclusion, such as personal goal setting [5]. People with ID increasingly live in community houses rather than in segregated institutions, which gives them more opportunity to participate in the community. This development can be typified as a shift towards a citizenship paradigm [6]. In the citizenship paradigm, relatives and professionals encourage the self-determination and personal development of people with ID [7]. The ‘new’ professional has become an ‘enabler’, a companion who enables a person with ID to participate in society and reach their full potential [8]. The dominant values underlying the citizenship paradigm are e.g. helping the person with ID to realize self-actualization and reach autonomy.

Another relevant development for people with ID is the improvement in health care over recent decades. Consequently, the life expectancy of people with ID has increased and they form an aging population. This epidemiological development is connected with growing incidences of life-limiting illnesses, such as progressive cancer, chronic cardiovascular diseases, chronic lung diseases and dementia [9–11]. As a result, professionals and relatives increasingly have to deal with people with ID who need end-of-life care. End-of-life care may start early on in the trajectory of a life-threatening illness and can be typified as multidisciplinary care aimed at enhancing the quality of life by assessing and relieving pain and other distressing symptoms, while taking account of the physical, psychological and spiritual needs of patients and their families. End-of-life care for people with ID is known to be particularly challenging due to their limited level of understanding and communication difficulties (e.g. [12, 13]). Several studies have indicated that ID care professionals have a lack of knowledge and expertise in end-of-life care, for example in pain and symptom management [14–16]. Todd interviewed ID care staff and found that although care staff are willing to provide end-of-life care for their clients (with whom they often have strong bonds), they can be very overwhelmed by caring for a dying person. This is amplified by a lack of knowledge and a lack of the kind of support that would enable them to provide this care properly [17, 18]. In an earlier study, we found that insufficient expertise among care staff within ID care services often prompts the decision to move a terminally ill client to another care setting [19].

End-of-life care may require a different care approach and attitude to what caregivers are used to. Kirkendall argues that when death is imminent, people with ID need an approach covering all aspects of end-of-life care, an approach that may not be naturally rooted in ID care services [20]. Thus far, it is unclear whether and how relatives and professionals change their care approach and attitudes when the death of a person with ID is imminent. To gain more insight into these processes, this paper will address the following research questions: do relatives, ID care staff and ID physicians perceive a shift in their care approach and attitudes when the death of a person with ID is imminent? And if so, what shifts in care approaches and attitudes do they perceive? And what values underlie these shifts?

## Methods

### Design

This study used a retrospective case study design. We held semi-structured interviews with professionals and relatives who were close to a person with ID at the end of life. In this way, end-of-life care of twelve recently deceased people with ID were reconstructed from different perspectives. Such a qualitative design is particularly well suited to comprehending complex issues.

This paper is part of a wider study set up to increase knowledge about end-of-life care for people with ID in the Netherlands. From the inductive analyses of the data it appeared that some major shifts in care approaches were taking place. These shifts are described in this paper. Another paper, based on the same qualitative data, describes another main theme emerging from the analyses, namely how care providers try to respect the autonomy of people with ID at the end of life [21].

### Participants

Twelve recently deceased people with ID were selected by the contact persons at ten ID care services organizations. In total, 45 professionals and relatives who were close to one of these recently deceased people were interviewed. Table 1 shows that the cases comprised six men and six women with different degrees of severity of ID. Half of them died of cancer. They reached a mean age of 53 years (range from 13 to 74). Six died in their own living environment, two in an intensive-care unit of their ID care service, three in a hospital and one in a hospice. Nine of the twelve people with ID lived in community houses and three in houses in a residential complex. All received care or support from an ID care service. All but one person died less than a year before the interview was held, allowing us to retrieve the participants’ recent memories. One person died two-and-a-half years before the time of the interview and was included because of his specific situation, dying in a hospice.

**Table 1:** General overview of the cases: the deceased people and the interviewees

	Relatives interviewed	Professional caregivers interviewed
Case 1: female, died from dementia, moderate intellectual disability	1 mentor	1 nurse
	(a friend)	1 ID physician
		1 psychologist
Case 2: female, died from metabolic disease, severe/profound intellectual disability	1 mother	2 social workers^1^
		1 nurse
		1 GP
Case 3: female, died from kidney failure and heart failure, mild intellectual disability	1 sister	1 social worker
	1 brother-in-law	1 GP
Case 4: male, died from cancer, mild intellectual disability	1 sister	1 social worker
	1 brother-in-law	1 nurse specialized in palliative care
		1 GP
Case 5: female, died from cancer, mild intellectual disability		1 social worker
		1 nurse
Case 6: male, died from cancer, severe/profound intellectual disability	1 sister	1 social worker
		1 ID physician
Case 7: male, died from metabolic disease, severe/profound intellectual disability	1 mother	1 social worker
		1 nurse
		1 ID physician
Case 8: male, died from cancer, mild/moderate intellectual disability	1 sister	2 social workers
		1 ID physician
Case 9: male, died from cancer, mild intellectual disability	2 sisters	1 social worker
		1 hospice coordinator
Case 10: female, died from dementia, moderate intellectual disability	1 brother	1 nurse
		1 ID physician
Case 11: male, died from frailty in old age, severe/profound intellectual disability	1 brother	1 social worker
		1 end-of-life care consultant^2^
Case 12 female, died from cancer, moderate intellectual disability	1 sister	2 social workers
		1 ID physician
Total	16	29

^1^In the Netherlands, social workers who work in ID care services provide support to people with ID in their daily lives. These social workers usually have an associate degree in socio-pedagogical support

^2^The end-of-life care consultant in this case was a nurse from the ID care service, specialized in end-of-life care, who advises teams of nurses and social workers on how to provide end-of-life care

### Data collection

Individual in-depth semi-structured interviews were held at the place of preference of the interviewees. Interviews were conducted by the first author and lasted one to two hours. Open questions were used, encouraging interviewees to describe the case in their own words. A topic list was used as a guide for formulating interview questions. The topic guide was developed in two stages: 1) the first draft of the topic guide was inspired by elements in the WHO definition of palliative care [22] and literature regarding end-of-life care for people with intellectual disabilities [23–25]. 2) This draft guide was discussed within a group of six experts working in the field of end-of-life care for people with intellectual disabilities, after which the guide was refined and finalized. The first question in the topic guide was what had made the strongest impression on the interviewee during the period of end-of-life care. Subsequent topics or questions concerned the care provided, communication with the person with ID, communication among professionals and between professionals and family, end-of-life decisions and dealing with loss. Table 2 presents an overview of the main topics. The order in which topics were discussed varied, depending on the course of the interview.

**Table 2:** Overview of the main interview topics

Opening question	What made the strongest impression on the interviewee during the period of end-of-life care?
Topic 1	Marking the start of palliative phase, communication about the diagnosis
Topic 2	Primary care process (e.g. pain, other physical symptoms, physical care, psychosocial well-being and spiritual well-being)
Topic 3	Decisions at the end of life
Topic 4	Communication with the person with ID
Topic 5	Autonomy of the person with ID
Topic 6	Transitions (e.g. changes in place of care or caregivers)
Topic 7	Communication and cooperation with relatives
Topic 8	Care for relatives (e.g. dealing with loss)
Topic 9	Fellow residents
Topic 10	The funeral
Topic 11	Aftercare
Topic 12	Care for carers (e.g. dealing with loss)

Participants were selected purposively in order to obtain a relatively diverse set of client cases with regard to age, living situation (own apartment, group home or residential home), place of death (own home, parents’ home, intensive care facility provided by an ID care service, hospital or hospice), kind of disease and severity of ID (mild, moderate or severe ID). In this way, a variety of situations could be taken into account in the study. The number of cases was not predetermined. Rather, data collection and analysis were conducted in a cyclical process in accordance with key principles of qualitative research [26]. Data saturation [27] was attained after twelve cases.

### Analysis

Interviews were taped and transcribed verbatim. Data were analyzed inductively, using elements of thematic analysis [28]. Important elements of thematic analysis are familiarizing with the data, generating initial codes, searching for themes, reviewing and defining themes and writing down the results. Thematic analysis was applied as follows: interviews were re-read and codes were ascribed to those text fragments potentially reflecting shifts and values. We used descriptive codes directly based on words that the interviewees used (such as *having two families*), as well as interpretative codes (such as *attentiveness*). Interview fragments with the same codes were constantly compared. Written memos were made concerning the interpretation of text and the relationships between codes throughout the analysis process. All authors were involved in the entire process from the analyses of interviews to the generation of concepts. All interviews were analyzed by the first author. To ensure reliability, each co-author individually analyzed all interviews for at least one case. Correspondence between interpretations and the original interviews was continuously verified and differences in analysis and interpretations were discussed. After the first author had discussed a third of the interviews with the co-authors, consensus was reached about the chosen codes and their interpretation. After coding all transcripts, potential themes were formulated. These themes were then reviewed in relation to the coded text fragments and transcripts. Finally, themes were refined and written down. MAXQDA 2007 was used to facilitate the analysis [29].

### Ethical consideration

All interviewees were mentally competent individuals and no interventions were performed. In such cases, no approval by an ethics committee is required in the Netherlands, according to the Medical Research Involving Human Subjects Act [30]. All respondents received a letter informing them of the aim of the study and gave informed consent. Study participation was voluntary. The responses were anonymous and non-traceable to individuals.

## Results

Relatives, care staff and ID physicians perceived several shifts in their care approaches and attitudes as the death of a person with ID approached. Five shifts were distinguished. They are described below, including the values behind these shifts. To illustrate the shifts, we present the cases of Josh, Eleanor and Joe (see Table 3; the names used in the case descriptions are not the subjects’ real names). We also discuss the extent to which they resemble the other cases studied.

**Table 3:** Cases illustrating the perspectives of care staff, physicians and relatives

*Josh* was a man aged 58, with moderate intellectual disability (Down syndrome). He died from dementia. Josh lived in a group home in the community.
*Eleanor* was a frail woman aged 74, with Parkinson’s disease and heart failure. Eleanor was in a wheelchair and had a severe intellectual disability. Eleanor lived in a group home in the community. She died in a hospital.
*Joe* was a man aged 40, with a severe intellectual disability and lung cancer. Joe lived in a small-scale living facility on the residential premises of an ID care service.

### From activating towards comfort care and taking over tasks

For care staff, providing care for a person with ID in their last phase in life generally meant a shift to a different care approach. Care staff were used to focusing on encouraging the person to undertake activities. However, as the terminal condition progressed and the person with ID became less and less able to participate in daily activities (such as day care, work and hobbies), care staff took over more and more daily tasks. Meanwhile, care staff increasingly put the emphasis on paying more loving attention to the person with ID and providing comfort care, such as holding hands, giving massages, offering favorite foods, listening to favorite music, having reassuring talks, or undertaking small favorite activities such as blowing bubbles or watching football. In the last stage in life, even regular care, such as showering or eating, was sometimes burdensome and new solutions needed to be found, such as reducing time taken showering, washing in a bath chair and offering favorite foods in a liquid form. Josh’s nurse described how she experienced the transition towards end-of-life care:

Josh’s Nurse: *‘There are no ‘musts’ anymore. It's more about letting go, letting Josh have a fine last week. Let him snuggle in bed. He doesn’t have to sit up if he doesn’t have the power to do so. But it was very difficult to let it all go. <> I often wondered: when am I going to take it all over from him? We have learned to encourage clients in all their daily activities and even encourage them to do more activities. Now you have to let go. You should ask yourself: who do I keep offering the activities for? <> We are not doing him a favor. Josh will never be the same person. <> This awareness came slowly, but it was very hard.’*

As Josh’s case illustrates, for most care staff, the shift from care as usual to end-of-life care implied a continuous search for a new balance between offering activities (perhaps adapted) and taking over tasks from the person with ID. This could be quite difficult, as it implied ‘letting go’ of parts of a care strategy that care staff identified with as being the essence of their work, namely expanding the activities and encouraging self-reliance.

For most relatives, it was particularly hard to see the decline in the health of their relative, particularly when this was a process of rapid deterioration. In some cases, the person ended up in a wheelchair and eventually in bed in just a matter of days, not being able to eat or drink properly. In other cases, the deterioration went more slowly, for example in the case of dementia or frailty due to old age. But even then, the person could become weaker quickly in the final weeks. Joe had cancer and his sister described how she experienced the shift:

Joe’s sister: ‘*Joe always liked to go outside. He had a custom-made bike and we often went out together. He could also really shake his head and say: “I don’t want to go out”. But I thought: he should go out, it’s good for his health, and he will see some different things.<> Then I heard of a woman who also had cancer and who complained that her friends would always take her out, but she had to recover for a week from these outings, and she actually did it more for them than for herself. I thought: why do I still take Joe out? Only to tell myself he is doing fine? Then I decided: if he doesn’t want to go, we won’t go. We don’t have to keep him in shape, he’s going to die. I have to look at his abilities and should not assume that shaking his head is just a gesture, it really means no.’*

Joe’s sister reflected on her activity planning with her brother and came to the conclusion that she needed to respond better to his signals. Like Joe’s sister, many relatives were used to encouraging their relative to be active and now increasingly had to let go of these expectations, adapting to what was still possible.

‘Being there’ and making more time available to be close to the ill person and subsequently ‘being responsive’ to the person’s real needs and possibilities were identified as values underlying the shift towards comfort care and taking over tasks.

### Interweaving of emotional and professional involvement becomes a struggle

The second shift dealt with the interweaving of the emotional and professional involvement of care staff with their clients. This interweaving had always been there, but increasingly became a struggle as the death of a client came near. In ID care services, many care staff members have built long-term caring relationships with their clients, which distinguishes this setting from many other healthcare settings. Their compassionate caring relationships were sometimes even described as a ‘special bond’, ‘love’ or ‘friendship’. The prospect of losing this client combined with the often hectic period of intense care for the dying client and their co-residents led to a rise in emotions that could be hard to deal with. Eleanor’s social worker described how she experienced her emotions:

Eleanor is in the hospital and she is expected to die soon. Her social worker and brother are visiting her as often as they can. The social worker is about to go on holiday. She is stressed about this: *‘I didn’t want Eleanor to be alone in the hospital. I found it hard to go home. <> Eleanor’s brother told me several times I could go home. Then I decided: I can only stay this long tonight, then I have to go home, I am going on holiday the next day. <> There are boundaries, I could not have stayed all night. I told myself: I need to transfer my care for Eleanor to others now. But that was very hard. I couldn’t get that act together. I was so deeply involved in her care. It was hard to decide to leave. <> So I wrote a card for Eleanor, for in her coffin and for her brother to wish him strength. And then I went home, to pack for my holiday. But I did not feel like holiday at all’.*

Joe’s social worker also talked about her last time with Joe:

*‘We always had a special bond. I was really fond of Joe. He was really one of my special clients. <> (in tears) In the team, we decided to care for him ourselves. He wanted to be with us. It felt like family. Caring for him at the end of his life was hard, but rewarding. <> I tried to keep in mind that he was my client, to help me cope. But I didn’t let him notice that, I was very involved with him. I didn’t want him to be in any distress.*

Like Eleanor’s and Joe’s social workers, many care staff members struggled to find ways to provide warm, tender end-of-life care but at the same time keep an emotional distance. In this process, most care staff members needed the emotional support of their colleagues and superiors. This period could be particularly intense for care staff members who were providing end-of-life care for the first time. A lack of experience could make care staff insecure and anxious about what was going to happen. Reflection was identified as a value for dealing with the interweaving of emotional and professional involvement; being able to reflect on your own emotions, capabilities and caring relationship helped care staff to find a balance between warm care and emotional distance.

### Symptom relief: stronger reliance on the joint interpretation of signals

Symptom relief became very important during end-of-life care. In particular it was often a challenge to identify pain, anxiety and other signals of distress in people with severe ID. Often these symptoms only became visible through small changes in behavior or signals such as grimaces or stretching the neck. These changes were not easily recognized. This meant that physicians now relied heavily on information from people closely involved with the person, mostly relatives and care staff. The case of Joe is an example of this:

Joe’s ID physician: *‘It took a while before we had the idea that Joe was indicating pain. <> The idea was that Joe would not have to suffer in any case. <> My role as a physician was mainly on demand. It’s the care staff who know him best, they pick up signals such as pain. Then they call me. <> But the identification of pain is largely done by the care staff and the family. I think in Joe’s case it was his sister who first said to me he’s in pain now. And over time everyone agreed that he was in pain. <> You could tell that Joe had pain by his moaning and grabbing. He used to clutch his chest or neck. And make groaning noises. And he would wince. He was also very affectionate towards his care staff and to his mother or anyone who was familiar for him at that time. Whether that expressed real pain I don’t know but I felt he was deteriorating. He sought more proximity and couldn’t be left alone.’*

Although many physicians were already used to incorporating the views of others in their regular care, the reliance on third-party information became more pronounced in end-of-life care. Symptom relief became a real team effort. The joint interpretation of signals could be a hurdle as relatives, care staff and physicians did not always proactively seek contact and did not always find a common language to talk about what they saw. Two values were essential in this shift: 1) ‘attentiveness’ to the person’s signals and expressions of needs, distress and pain, often best done by permanent care staff and relatives who had a life-long relationship with the person, and 2) ‘openness to cooperation and building a shared understanding’ of the interpretation of signals and expressions.

### Magnified dependency and responsibility in medical decisions

In most of the cases studied, many medical decisions needed to be made during the end-of-life care, for example about life-prolonging treatments, tube feeding, providing oxygen or pain medication by infusion pump. In most cases, the role of the people with ID themselves was unclear in the decision-making process, especially for people with more severe ID. Physicians usually discussed medical information with relatives and mentors, and tried to come to shared decisions with them. Relatives and mentors often felt overwhelmed by the dependency of the person with ID, which seemed magnified during end-of-life care, in particular concerning decisions about prolonging treatment, tube feeding or palliative sedation. The case of Josh is an example:

Josh’s physician and mentor are discussing whether they should place a tube to facilitate Josh’s feeding. Josh would need to go to the hospital to have the tube placed. Josh’s mentor: *‘I said I don’t want him to go to the hospital to get the tube. If Josh is in the hospital he gets very scared by all those white coats, you wouldn’t make him happy by doing that. <> So the doctor told me they may be able to do it in the ID care center. But then again, the tube would only prolong his life a little bit. Would that be right? I wanted to know, am I doing him harm if he doesn’t get the tube? Will he starve to death and feel awful? The doctor said that that was not going to happen, he gave me the confirmation that they would relieve his suffering. That was what I needed to hear. I wanted to be able to look at myself in the mirror and tell myself that I did not do anything very burdensome to him at the end of his life.’*

Like Josh’s mentor, many relatives were insecure, often because their sick relative was unable to adequately indicate what his or her wishes were. They had to deal with the emotions of feeling highly responsible, and sometimes even felt they were deciding about the other person’s life and death. Four values were behind this shift: 1) ‘responsibility’ for taking joint decisions in the best interests of the person, 2) ‘attentiveness’ to the person’s wishes, 3) relatives’ ‘reflection’ on their own emotions, and 4) ‘openness to cooperation and making shared decisions’.

### Growing awareness of having two ‘families’

In most cases, the imminence of the end of life led to an intensified caring relationship between relatives and care staff. Relatives visited more often, became more involved in the caring process and sometimes even gave care jointly with care staff. Some relatives and care staff were easily able to coordinate their activities and make clear agreements, aiming for the same goal of being there and providing the best possible warm, comforting care for the sick person. In these cases, relatives were often full of praise for the support they received from the professionals in the ID care service. However, it was not always easy for relatives to deal with the professionals during this emotional period. In some cases, cooperation did not run smoothly and relatives and care staff had different perceptions of what good end-of-life care entailed. In one case, the relative experienced the close involvement and advice of the care staff member as an infringement of her privacy and her rights as a representative. Other relatives struggled initially in cooperating with the care staff, but eventually found ways to properly establish shared care. As Joe’s sister declared: ‘I came to realize he had “two families”’:

Joe’s sister: *’I drove back from my holiday because I had received a call that Joe was rapidly becoming weaker <>. I thought, gosh, if he dies, then there will only be six of us at his funeral. Well, we all love him, not a lot of people need to be there. <> And while driving I thought: But actually his family is much bigger than our family. Joe has two families. Our family* (the relatives) *sees him as a man with an intellectual disability, but at his home at the ID care service he has another family who see him as Joe, as a person with a sense of humor and jokes. They* (the care staff) *talk in a very different way to him than we did. That was good to realize.<> After Joe died, it felt like we* (the relatives and care staff) *were really* one *family, all care was finished, and we were there for each other.’*

‘Openness to cooperation and sharing’ the care was an important value to accompany the intensified presence of two ‘families’.

## Discussion

### Specifics of end-of-life care for people with ID

Although some of the shifts found in this study may also apply to other client groups and care settings, several aspects seem particularly relevant for the end-of-life care for people with ID. First, ID care staff, more than professionals in other healthcare sectors, are trained to activate their client and are used to doing this. At the end of life, their usual strong focus on improving the client’s quality of life by maximizing the activation and participation of people with ID in society has to change to a strong focus on the quality of life through comfort care, taking over tasks and good symptom relief. Second, the increasing focus on the identification and relief of symptoms is particularly challenging as verbal communication with a person with ID is often hampered. Hence, professionals and relative rely strongly on their joint interpretation of signals of distress in the person with ID. This can be problematic as ID care staff, and in particular social workers, are known to have a shortage of knowledge and skills regarding e.g. the use of instruments to measure pain or other symptoms at the end of life [15]. Third, another typical factor is that decision making about, for instance, whether to continue or forgo life-prolonging treatments at the end of life is particularly complex because people with ID often have difficulties with clearly expressing personal care needs or wishes. Relatives feel highly responsible as a ‘proxy’, and feel that their relative with ID becomes increasingly dependent on them. Fourth, the fact that both the family and the care staff at the ID care service are often deeply emotionally involved over a long period of time is quite specific to the end-of-life care for people with ID. Among care staff, the interweaving of emotional and professional involvement often leads to struggles in trying to achieve a balance between warm care and professional distance.

### Comparison with existing literature

The impact on relatives of ‘having to let go’ and being overwhelmed by the feeling of being responsible for ‘deciding for somebody else’ has been described earlier in papers, for example in our and other studies on end-of-life care for people with ID [21, 31], and also in papers on children with cancer [32]. Care staff, the other ‘family’ in our study, experienced a rise in emotions when they had to let go of a client they had often taken care of for a long period of time. Similar emotions during the end-of-life care among ID care staff were also found by Wiese [33]. These ‘family-like’ relationships between care staff and clients have also been observed in other settings, such as dementia care [34]. In a nursing home study, care staff pinpointed the loss of the close attachment to their client as the biggest challenge to overcome in the transition to end-of-life care [35]. Yet for care staff in ID care services ‘letting go’ might be even more intense, as providing end-of-life care is not regular care for them. Generally, ID care staff serve clients of all ages. Indeed, many care staff in our study (in particular inexperienced staff) struggled to channel their feelings in order to foster professionalism and secure the provision of tender end-of-life care. These struggles may jeopardize the caring relationships between care staff and clients. Wagemans [36] also found such struggles and described them as ‘balancing involvement and distance’. The ID care staff in her study suggested that keeping too much distance might hamper good care. The struggles seem to reveal an inner conflict within ID care staff members, which calls for more attention than is currently given in ID care services.

Having ‘two families’ (relatives and care staff) come together and jointly provide care for you as a dying person with ID can lead to warm, loving care in the proximity of the very people who are most important to you and who know you and your needs best. Yet, if perceptions of what constitutes good care differ and communication fails, the good intentions to provide joint care may turn into a burden and source of distress for the dying person. Problems in the joint provision of end-of-life care between relatives and care staff are not unique to ID care: they were also found in nursing homes and care homes [34, 37, 38]. A difference is that people with ID have generally lived much longer in their care setting than people in care homes or nursing homes. Relationships between relatives and ID care services often go back decades, making the comparison of ‘two families’ much stronger. Moreover, in contrast to most nursing homes, ID care staff often lack expertise and experience in end-of-life care [15, 39, 40], which puts them more on a level with relatives in terms of end-of-life care expertise. Care staff may be just as insecure about what to do as relatives. This can put existing care relationships between relatives and care staff under pressure and may jeopardize good end-of-life care. ID care services should pay timely attention to this relationship, and to the expertise of their staff.

### Values underlying the shifts at the end of life of people with ID

Six values were behind the shifts in end-of-life care for people with ID: 1) ‘Being there’ and making extra time available to be close to the sick person. Being there was a prerequisite for the second value. 2) ‘being responsive’ to the person’s real needs and possibilities. Being there and being responsive both refer to the acknowledgment of what the person is capable of and acting on both the durable and the changing capabilities. ‘Being there’ has been recognized as an important theme in other end-of-life care studies, e.g. of people with ID [41], and children with cancer [42]. ‘Reflection’ on your own emotions, caring capabilities and the relationship with the person with ID. Reflection proved particularly helpful in achieving a balance between the emotional involvement and professional distance of care staff, and in enabling relatives to channel their emotions when confronted with medical decisions. 4) ‘Attentiveness’ to the person’s needs and wishes and expressions of distress and pain. Attentiveness is generally found to be important for end-of-life care. E.g. Leget stressed the importance of listening attentively to dying patients in order to unravel their *real* needs and questions (‘inner space’; [43]). Unravelling the real needs and distress can be extremely complex in people who have ID, and yet is therefore so very essential. In particular, people with severe ID rely heavily on deep, long-lasting relationships with close caregivers. 5) ‘Responsibility’ for taking joint decisions in the best interests of the person with ID, particularly evident among relatives who felt a great sense of responsibility for taking the right decision. 6) Finally, ‘openness to cooperation and sharing’ comprised the intensified cooperation among professionals and between relatives and professionals in providing good end-of-life care: e.g. being able to communicate adequately about the person’s signals and needs, the openness required to take joint medical decisions and the openness of the ‘two families’ to enable the joint provision of care. The six aforementioned values are all highly relational. They are associated with a care ethics perspective, which recognizes that all care is relational [44]. Care for people with ID inevitably builds on relationships, as helping people to participate and get a job, for instance, requires caregivers to have many relational qualities. At the end of life, the value of good caring relationships seems to increase. Three caring relationships need to be fostered: the relationship with the person with ID, relationships among professionals and the relationship between relatives and professionals.

### Strengths and weaknesses

We were able to reconstruct the story of twelve deceased people with ID by using a multiperspective design, incorporating the viewpoints of all the people most closely involved. A limitation is that we conducted this study retrospectively, so the experiences of the interviewees may have been subject to a recall bias. On the other hand, retrospective studies make people’s statements less susceptible to day-to-day emotions.

This study did not explore shifts in the end-of-life care approaches to people without ID. Future research could reveal the extent to which the shifts found in this study match shifts in care approaches among other client groups in long-term care settings, such as people with dementia and residents in nursing homes and elderly care homes.

## Conclusion

As the death of a person with ID comes near, several shifts are perceived: care staff and relatives have to let go of their usual care strategy aimed at activation; the interweaving of emotional and professional involvement becomes a challenge for care staff; the joint interpretation of signals expressing distress becomes increasingly important; the dependency of the person with ID rises in the eyes of relatives (their ‘proxy’ decision makers), in particular with regard to medical decisions; and at the end of life it becomes increasingly evident that the ID care staff have also become a ‘family’ for the client. Highly relational values are behind these shifts: ‘being there’ for the person with ID, ‘being responsive’ to the person’s needs, ‘reflection’ on their own emotions and caring relationships, ‘attentiveness’ to the ID person’s wishes and expressions of distress, ‘responsibility’ for taking joint decisions in the best interests of the person, and ‘openness to cooperation and sharing’ the care with others.

### Practice implications

End-of-life care for people with ID requires a different care approach and attitude to participation-focused care. It requires an allowance for decreasing expectations as regards activities and skill acquirement, and an increase in teamwork featuring intensified comforting care, symptom management and medical decision making. Cooperation among close caregivers is fundamental, as is emotional support for care staff. As more and more people with ID will need end-of-life care in the future, ID care services should be better prepared. With regard to supportive care for carers, services should invest particularly in three areas. First, sufficient emotional support should be given for care staff who struggle to find a balance between their emotional involvement and professional distance. Informal support from colleagues alone may not be sufficient. These inner conflicts require professional support sessions, including time for intrapersonal and interpersonal reflection. Secondly, improving care staff’s level of expertise in end-of-life care is necessary, as care staff can be inexperienced and insecure in dealing with both clients and relatives. Some end-of-life care training interventions for ID care staff have already been developed (e.g. [45–48]). Thirdly, the relationship between relatives and care staff needs attention, as good cooperation is essential for the dying person with ID. Moreover, relatives need the support of professionals as they go through the process of letting go and have to make difficult decisions. Care staff need to learn how to build strong, good working relationships with relatives, preferably at a much earlier stage in the lives of people with ID as emotions usually run high when death is imminent.
